# [(4*E*,11*E*)-5,7,12,14-Tetra­benzyl-7,14-dimethyl-1,4,8,11-tetra­aza­cyclo­tetra­deca-4,11-diene]copper(II) bis(per­chlorate)

**DOI:** 10.1107/S1600536811042796

**Published:** 2011-10-22

**Authors:** Tapashi G. Roy, Saroj K. S. Hazari, Babul C. Nath, Seik Weng Ng, Edward R. T. Tiekink

**Affiliations:** a Department of Chemistry, University of Chittagong, Chittagong-4331, Bangladesh; b Department of Chemistry, University of Malaya, 50603 Kuala Lumpur, Malaysia; c Chemistry Department, Faculty of Science, King Abdulaziz University, PO Box 80203 Jeddah, Saudi Arabia

## Abstract

The complete cation in the title compound, [Cu(C_40_H_48_N_4_)](ClO_4_)_2_, is generated by the operation of a crystallographic centre of inversion. The Cu^II^ ion exists in a tetra­gonally distorted *trans*-N_4_O_2_ coordination geometry defined by the four N atoms of the macrocyclic ligand and two weakly bound perchlorate-O atoms from two anions. The N—H atoms form intra­molecular N—H⋯O(perchlorate) hydrogen bonds. Disorder was resolved in the –CH_2_–NH– portion of the macrocycle with the major component having a site-occupancy factor of 0.570 (6).

## Related literature

For background to the synthesis, characterization, kinetic studies and biological activities of 14-membered methyl-substituted tetra­aza­macrocyclic ligands, their *N*-substituted derivatives and their metal complexes, see: Hazari *et al.* (2008 ▶).
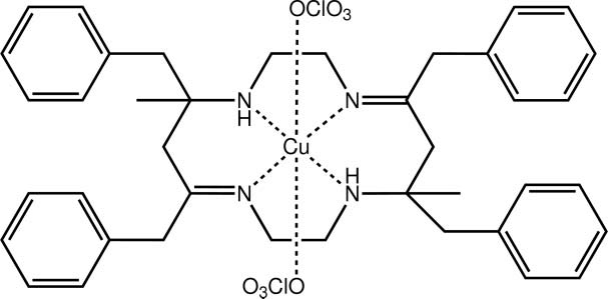


## Experimental

### 

#### Crystal data

[Cu(C_40_H_48_N_4_)](ClO_4_)_2_
*M* *_r_* = 847.26
Monoclinic, 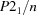

*a* = 10.1170 (3) Å
*b* = 16.6017 (4) Å
*c* = 11.9910 (3) Å
β = 108.818 (3)°
*V* = 1906.35 (9) Å^3^
*Z* = 2
Mo *K*α radiation
μ = 0.77 mm^−1^
*T* = 100 K
0.30 × 0.25 × 0.20 mm

#### Data collection

Agilent Technologies SuperNova Dual diffractometer with Atlas detector
Absorption correction: multi-scan (*CrysAlis PRO*; Agilent, 2010 ▶) *T*_min_ = 0.792, *T*_max_ = 1.000
9806 measured reflections
4253 independent reflections
3707 reflections with *I* > 2σ(*I*)
*R*_int_ = 0.024

#### Refinement

*R*[*F* ^2^ > 2σ(*F* ^2^)] = 0.059
*wR*(*F* ^2^) = 0.145
*S* = 1.08
4253 reflections
251 parameters
15 restraints
H-atom parameters constrained
Δρ_max_ = 0.64 e Å^−3^
Δρ_min_ = −1.08 e Å^−3^

### 

Data collection: *CrysAlis PRO* (Agilent, 2010 ▶); cell refinement: *CrysAlis PRO*; data reduction: *CrysAlis PRO*; program(s) used to solve structure: *SHELXS97* (Sheldrick, 2008 ▶); program(s) used to refine structure: *SHELXL97* (Sheldrick, 2008 ▶); molecular graphics: *ORTEP-3* (Farrugia, 1997 ▶) and *DIAMOND* (Brandenburg, 2006 ▶); software used to prepare material for publication: *publCIF* (Westrip, 2010 ▶).

## Supplementary Material

Crystal structure: contains datablock(s) global, I. DOI: 10.1107/S1600536811042796/hb6449sup1.cif

Structure factors: contains datablock(s) I. DOI: 10.1107/S1600536811042796/hb6449Isup2.hkl

Additional supplementary materials:  crystallographic information; 3D view; checkCIF report

## Figures and Tables

**Table 1:** Selected bond lengths (Å)

Cu—N1	2.032 (4)
Cu—N2	1.977 (2)
Cu—O1	2.662 (2)

**Table 2:** Hydrogen-bond geometry (Å, °)

*D*—H⋯*A*	*D*—H	H⋯*A*	*D*⋯*A*	*D*—H⋯*A*
N1—H1⋯O1^i^	0.88	2.39	2.940 (5)	121
N1′—H1′⋯O2	0.88	2.29	3.104 (4)	153

Symmetry code: (i) 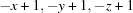.

**Fig. 1.**
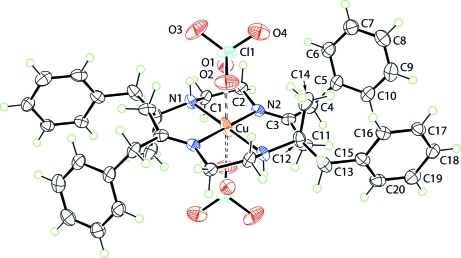
The molecular structure of (I) showing displacement ellipsoids at the 50% probability level. Only the major component of the disordered residue is shown. Unlabelled atoms are generated by the symmetry operation (1–x, 1–y, 1–z).

## supplementary crystallographic information

### Comment

The title complex, (I), was investigated in continuation of studies of the synthesis, characterization and biological activities of methyl substituted tetraazamacrocyclic ligands and their metal complexes (Hazari *et al.*, 2008).

The structure of (I), Fig. 1, features a tetragonally distorted N_4_O_2_ donor set about a Cu^II^ atom, Table 1. The N-donor atoms are derived from the (4*E*,11*E*)-5,7,12,14-tetrabenzyl-7,14-dimethyl- 1,4,8,11-tetraazacyclotetradeca-4,11-diene macrocyclic ligand, and the O-donors are derived from two perchlorate anions. The complex is centrosymmetric. A *C*-*meso*, *N*-*meso* configuration is found in (I). With reference to the six-membered chelate ring, the benzyl and methyl groups equatorially and axially orientated, respectively. The dihedral angle formed between the benzene rings is 44.22 (16) Å. Each N—H atom of the disordered —CH_2_—NH— residue forms an intramolecular N—H···O hydrogen bond with a perchlorate-O atom, Table 2, *i.e*. to either side of the CuN_4_ plane. The competition between the formation of these alternate hydrogen bonds provides a rationale for the observed disorder.

### Experimental

The macrocyclic ligand as its hydroperchloric acid salt (0.783 g, 1.0 mmol) was suspended in methanol (20 ml). Copper(II) perchlorate hexahydrate (0.370 g, 1.0 mmol) was dissolved in methanol (30 ml) and then was mixed with the suspension of the ligand salt. The mixture was refluxed for 3 h and a clear violet solution evolved. The solution was filtered and kept at room temperature. After 24 h, violet crystals of the complex were observed. The crystals were separated by filtration, washed with dry ethanol, followed by diethylether and dried in a vacuum desiccator over silica gel. *M*.pt. 510–512 K. Yield 45%. Anal. Calc. for [Cu(C_40_H_48_N_4_)](ClO_4_)_2_ C, 56.77; H, 5.56; N, 6.62; Cu, 7.51%. Found: C, 56.56; H, 5.53; N, 6.75; Cu, 7.35%. FT—IR (KBr, cm^-1^) 3220 ν(N—H), 3028 ν(Ar—H), 2980 ν(C—H), 1650 ν(C═N), 1375 ν(CH_3_), 1185 ν(C—C), 1126 ν(ClO_4_), 710 ν(ArC—H)), 488 ν(Cu—N). The light-purple prisms were prepared by slow evaporation of a methanol solution of the complex.

### Refinement

One portion of the macrocycle, *i.e*. the –CH_2_–NH– portion, is disordered over two positions, with the major component having a site occupancy factor = 0.570 (6). The pair of Cu—N distances were tightly restrained to within 0.005 Å of each other, as were the C—N and C_disordered_—C_ordered_ distances. The H-atoms were placed in calculated positions (N—H = 0.88 Å and C—H = 0.95–0.99 Å) and were included in the refinement in the riding model approximation, with *U*_iso_(H) set to 1.2–1.5*U*_equiv_(N,*C*). The maximum and minimum residual electron density peaks of 0.64 and 1.08 e Å^-3^, respectively, were located 1.00 Å and 0.94 Å from the O4 and Cu atoms, respectively.

### Crystal data

**Table:**

[Cu(C_40_H_48_N_4_)](ClO_4_)_2_	*F*(000) = 886
*M**_r_* = 847.26	*D*_x_ = 1.476 Mg m^−^^3^
Monoclinic, *P*2_1_/*n*	Mo *K*α radiation, λ = 0.71073 Å
Hall symbol: -P 2yn	Cell parameters from 4647 reflections
*a* = 10.1170 (3) Å	θ = 2.3–29.3°
*b* = 16.6017 (4) Å	µ = 0.77 mm^−^^1^
*c* = 11.9910 (3) Å	*T* = 100 K
β = 108.818 (3)°	Prism, light-purple
*V* = 1906.35 (9) Å^3^	0.30 × 0.25 × 0.20 mm
*Z* = 2	

### Data collection

**Table:**

Agilent Technologies SuperNova Dual diffractometer with Atlas detector	4253 independent reflections
Radiation source: SuperNova (Mo) X-ray Source	3707 reflections with *I* > 2σ(*I*)
Mirror	*R*_int_ = 0.024
Detector resolution: 10.4041 pixels mm^-1^	θ_max_ = 27.5°, θ_min_ = 2.3°
ω scan	*h* = −11→12
Absorption correction: multi-scan (*CrysAlis PRO*; Agilent, 2010)	*k* = −21→16
*T*_min_ = 0.792, *T*_max_ = 1.000	*l* = −15→15
9806 measured reflections	

### Refinement

**Table:**

Refinement on *F*^2^	Primary atom site location: structure-invariant direct methods
Least-squares matrix: full	Secondary atom site location: difference Fourier map
*R*[*F*^2^ > 2σ(*F*^2^)] = 0.059	Hydrogen site location: inferred from neighbouring sites
*wR*(*F*^2^) = 0.145	H-atom parameters constrained
*S* = 1.08	*w* = 1/[σ^2^(*F*_o_^2^) + (0.0495*P*)^2^ + 4.3222*P*] where *P* = (*F*_o_^2^ + 2*F*_c_^2^)/3
4253 reflections	(Δ/σ)_max_ < 0.001
251 parameters	Δρ_max_ = 0.64 e Å^−^^3^
15 restraints	Δρ_min_ = −1.08 e Å^−^^3^

### Special details

**Table:**

Geometry. All e.s.d.'s (except the e.s.d. in the dihedral angle between two l.s. planes) are estimated using the full covariance matrix. The cell e.s.d.'s are taken into account individually in the estimation of e.s.d.'s in distances, angles and torsion angles; correlations between e.s.d.'s in cell parameters are only used when they are defined by crystal symmetry. An approximate (isotropic) treatment of cell e.s.d.'s is used for estimating e.s.d.'s involving l.s. planes.
Refinement. Refinement of *F*^2^ against ALL reflections. The weighted *R*-factor *wR* and goodness of fit *S* are based on *F*^2^, conventional *R*-factors *R* are based on *F*, with *F* set to zero for negative *F*^2^. The threshold expression of *F*^2^ > σ(*F*^2^) is used only for calculating *R*-factors(gt) *etc*. and is not relevant to the choice of reflections for refinement. *R*-factors based on *F*^2^ are statistically about twice as large as those based on *F*, and *R*- factors based on ALL data will be even larger.

### Fractional atomic coordinates and isotropic or equivalent isotropic displacement parameters (Å^2^)

**Table:**

	*x*	*y*	*z*	*U*_iso_*/*U*_eq_	Occ. (<1)
Cu	0.5000	0.5000	0.5000	0.0368 (2)	
Cl1	0.85496 (8)	0.59569 (5)	0.64594 (7)	0.0320 (2)	
O1	0.7221 (2)	0.59629 (15)	0.5522 (2)	0.0349 (6)	
O2	0.8616 (3)	0.52613 (18)	0.7184 (3)	0.0520 (8)	
O3	0.9641 (3)	0.5963 (2)	0.5963 (3)	0.0660 (10)	
O4	0.8647 (3)	0.66693 (18)	0.7171 (2)	0.0492 (7)	
N1	0.4748 (5)	0.4782 (3)	0.6587 (3)	0.0245 (10)	0.570 (6)
H1	0.4019	0.4462	0.6445	0.029*	0.570 (6)
N2	0.6240 (3)	0.40479 (15)	0.5342 (2)	0.0239 (5)	
C1	0.6015 (5)	0.4282 (3)	0.7295 (4)	0.0277 (11)	0.570 (6)
H1A	0.6845	0.4631	0.7610	0.033*	0.570 (6)
H1B	0.5825	0.4011	0.7964	0.033*	0.570 (6)
C2	0.6264 (3)	0.36629 (19)	0.6456 (3)	0.0261 (7)	
H2A	0.7181	0.3401	0.6825	0.031*	0.570 (6)
H2B	0.5533	0.3242	0.6294	0.031*	0.570 (6)
H2C	0.7195	0.3726	0.7058	0.031*	0.430 (6)
H2D	0.6056	0.3081	0.6334	0.031*	0.430 (6)
C3	0.7012 (3)	0.3815 (2)	0.4734 (3)	0.0257 (6)	
C4	0.7948 (3)	0.3080 (2)	0.4994 (3)	0.0282 (7)	
H4A	0.8072	0.2883	0.5800	0.034*	
H4B	0.8878	0.3221	0.4943	0.034*	
C5	0.7277 (3)	0.2425 (2)	0.4096 (3)	0.0281 (7)	
C6	0.6296 (4)	0.1915 (2)	0.4294 (3)	0.0352 (8)	
H6	0.6083	0.1955	0.5008	0.042*	
C7	0.5617 (4)	0.1344 (2)	0.3456 (4)	0.0399 (9)	
H7	0.4942	0.0999	0.3601	0.048*	
C8	0.5919 (4)	0.1277 (2)	0.2413 (3)	0.0379 (8)	
H8	0.5445	0.0892	0.1836	0.045*	
C9	0.6922 (4)	0.1776 (2)	0.2218 (3)	0.0374 (8)	
H9	0.7144	0.1728	0.1510	0.045*	
C10	0.7604 (3)	0.2349 (2)	0.3059 (3)	0.0309 (7)	
H10	0.8294	0.2687	0.2923	0.037*	
C11	0.7003 (4)	0.4281 (2)	0.3652 (3)	0.0316 (7)	
H11A	0.7518	0.3964	0.3226	0.038*	
H11B	0.7527	0.4789	0.3911	0.038*	
C12	0.5570 (4)	0.4493 (2)	0.2784 (3)	0.0303 (7)	
C13	0.5729 (4)	0.4799 (2)	0.1621 (3)	0.0354 (8)	
H13A	0.4791	0.4937	0.1074	0.042*	
H13B	0.6288	0.5301	0.1784	0.042*	
C14	0.4581 (3)	0.3778 (3)	0.2546 (3)	0.0391 (9)	
H14A	0.3668	0.3939	0.2004	0.059*	
H14B	0.4477	0.3591	0.3288	0.059*	
H14C	0.4962	0.3341	0.2191	0.059*	
C15	0.6410 (4)	0.42125 (19)	0.1011 (3)	0.0280 (7)	
C16	0.5637 (3)	0.36046 (19)	0.0285 (3)	0.0268 (7)	
H16	0.4664	0.3561	0.0163	0.032*	
C17	0.6273 (3)	0.3066 (2)	−0.0260 (3)	0.0287 (7)	
H17	0.5736	0.2650	−0.0742	0.034*	
C18	0.7686 (4)	0.3126 (2)	−0.0109 (3)	0.0316 (7)	
H18	0.8120	0.2756	−0.0485	0.038*	
C19	0.8455 (4)	0.3731 (2)	0.0598 (3)	0.0366 (8)	
H19	0.9421	0.3784	0.0698	0.044*	
C20	0.7815 (3)	0.42676 (17)	0.1166 (2)	0.0331 (8)	
H20	0.8358	0.4674	0.1665	0.040*	
N1'	0.5452 (3)	0.49931 (17)	0.6741 (2)	0.0245 (10)	0.43
H1'	0.6332	0.5119	0.7103	0.029*	0.430 (6)
C1'	0.5178 (3)	0.40711 (17)	0.6846 (2)	0.0277 (11)	0.43
H1'1	0.5276	0.3925	0.7670	0.033*	0.430 (6)
H1'2	0.4230	0.3922	0.6333	0.033*	0.430 (6)

### Atomic displacement parameters (Å^2^)

**Table:**

	*U*^11^	*U*^22^	*U*^33^	*U*^12^	*U*^13^	*U*^23^
Cu	0.0564 (4)	0.0281 (3)	0.0199 (3)	0.0258 (3)	0.0039 (3)	0.0029 (2)
Cl1	0.0177 (4)	0.0351 (4)	0.0413 (4)	−0.0034 (3)	0.0068 (3)	0.0122 (4)
O1	0.0232 (12)	0.0389 (14)	0.0379 (13)	0.0015 (10)	0.0034 (10)	−0.0016 (11)
O2	0.0465 (17)	0.0398 (15)	0.0623 (19)	−0.0023 (13)	0.0074 (14)	0.0250 (14)
O3	0.0303 (15)	0.092 (3)	0.087 (2)	−0.0031 (16)	0.0339 (16)	0.013 (2)
O4	0.0510 (17)	0.0442 (16)	0.0411 (15)	−0.0104 (13)	−0.0008 (13)	0.0021 (13)
N1	0.023 (2)	0.022 (2)	0.0222 (18)	0.0091 (17)	−0.0004 (19)	0.0010 (15)
N2	0.0225 (12)	0.0202 (12)	0.0252 (12)	0.0015 (10)	0.0024 (10)	0.0019 (10)
C1	0.025 (2)	0.034 (3)	0.026 (2)	0.013 (2)	0.0118 (18)	0.012 (2)
C2	0.0249 (15)	0.0244 (15)	0.0294 (16)	0.0057 (12)	0.0091 (13)	0.0081 (13)
C3	0.0182 (14)	0.0273 (16)	0.0282 (15)	−0.0013 (12)	0.0030 (12)	0.0037 (13)
C4	0.0182 (14)	0.0398 (18)	0.0266 (15)	0.0081 (13)	0.0075 (12)	0.0064 (14)
C5	0.0245 (15)	0.0303 (17)	0.0304 (16)	0.0158 (13)	0.0100 (13)	0.0096 (14)
C6	0.0354 (19)	0.0325 (18)	0.0418 (19)	0.0103 (15)	0.0183 (16)	0.0068 (16)
C7	0.038 (2)	0.0305 (18)	0.054 (2)	0.0072 (15)	0.0194 (18)	0.0029 (17)
C8	0.039 (2)	0.0277 (17)	0.042 (2)	0.0112 (15)	0.0063 (16)	0.0020 (16)
C9	0.042 (2)	0.039 (2)	0.0307 (17)	0.0218 (16)	0.0109 (15)	0.0106 (16)
C10	0.0245 (16)	0.0341 (18)	0.0361 (17)	0.0140 (13)	0.0127 (13)	0.0116 (15)
C11	0.0306 (17)	0.0320 (17)	0.0278 (16)	−0.0083 (14)	0.0033 (13)	0.0067 (14)
C12	0.0353 (18)	0.0277 (16)	0.0266 (15)	0.0127 (14)	0.0081 (13)	0.0019 (13)
C13	0.051 (2)	0.0247 (16)	0.0234 (16)	0.0058 (15)	0.0021 (15)	0.0004 (13)
C14	0.0204 (16)	0.066 (3)	0.0304 (17)	−0.0019 (16)	0.0080 (13)	−0.0073 (18)
C15	0.0372 (18)	0.0224 (15)	0.0204 (14)	0.0005 (13)	0.0035 (13)	0.0043 (12)
C16	0.0258 (15)	0.0268 (16)	0.0243 (15)	−0.0018 (13)	0.0030 (12)	0.0009 (13)
C17	0.0320 (17)	0.0268 (16)	0.0258 (15)	−0.0046 (13)	0.0072 (13)	−0.0018 (13)
C18	0.0318 (17)	0.0381 (19)	0.0268 (16)	−0.0006 (15)	0.0120 (14)	0.0002 (15)
C19	0.0298 (17)	0.051 (2)	0.0269 (16)	−0.0090 (16)	0.0058 (14)	−0.0010 (16)
C20	0.0376 (19)	0.0343 (18)	0.0235 (15)	−0.0129 (15)	0.0046 (14)	−0.0011 (14)
N1'	0.023 (2)	0.022 (2)	0.0222 (18)	0.0091 (17)	−0.0004 (19)	0.0010 (15)
C1'	0.025 (2)	0.034 (3)	0.026 (2)	0.013 (2)	0.0118 (18)	0.012 (2)

### Geometric parameters (Å, °)

**Table:**

Cu—N1	2.032 (4)	C7—H7	0.9500
Cu—N2	1.977 (2)	C8—C9	1.386 (6)
Cu—N2^i^	1.977 (2)	C8—H8	0.9500
Cu—N1'^i^	1.988 (2)	C9—C10	1.396 (5)
Cu—N1'	1.988 (2)	C9—H9	0.9500
Cu—N1^i^	2.032 (4)	C10—H10	0.9500
Cu—O1^i^	2.662 (2)	C11—C12	1.528 (5)
Cu—O1	2.662 (2)	C11—H11A	0.9900
Cl1—O3	1.413 (3)	C11—H11B	0.9900
Cl1—O2	1.433 (3)	C12—N1^i^	1.510 (5)
Cl1—O4	1.443 (3)	C12—C14	1.519 (5)
Cl1—O1	1.447 (2)	C12—C13	1.541 (5)
N1—C12^i^	1.510 (5)	C12—N1'^i^	1.582 (4)
N1—C1	1.534 (4)	C13—C15	1.511 (5)
N1—H1	0.8800	C13—H13A	0.9900
N2—C3	1.287 (4)	C13—H13B	0.9900
N2—C2	1.475 (4)	C14—H14A	0.9800
C1—C2	1.515 (5)	C14—H14B	0.9800
C1—H1A	0.9900	C14—H14C	0.9800
C1—H1B	0.9900	C15—C20	1.376 (4)
C2—C1'	1.489 (4)	C15—C16	1.396 (4)
C2—H2A	0.9900	C16—C17	1.383 (5)
C2—H2B	0.9900	C16—H16	0.9500
C2—H2C	0.9900	C17—C18	1.385 (5)
C2—H2D	0.9900	C17—H17	0.9500
C3—C4	1.514 (4)	C18—C19	1.381 (5)
C3—C11	1.509 (4)	C18—H18	0.9500
C4—C5	1.527 (5)	C19—C20	1.400 (5)
C4—H4A	0.9900	C19—H19	0.9500
C4—H4B	0.9900	C20—H20	0.9500
C5—C6	1.383 (5)	N1'—C1'	1.5679
C5—C10	1.392 (4)	N1'—C12^i^	1.582 (4)
C6—C7	1.390 (5)	N1'—H1'	0.8800
C6—H6	0.9500	C1'—H1'1	0.9900
C7—C8	1.385 (5)	C1'—H1'2	0.9900
			
O1—Cu—N1	103.76 (15)	H4A—C4—H4B	108.3
O1—Cu—N2	90.01 (9)	C6—C5—C10	119.3 (3)
N2—Cu—N2^i^	180	C6—C5—C4	119.7 (3)
N2—Cu—N1'^i^	97.92 (11)	C10—C5—C4	121.0 (3)
N2^i^—Cu—N1'^i^	82.08 (11)	C5—C6—C7	120.5 (3)
N2—Cu—N1'	82.08 (11)	C5—C6—H6	119.7
N2^i^—Cu—N1'	97.92 (11)	C7—C6—H6	119.7
N1'^i^—Cu—N1'	180	C8—C7—C6	120.4 (4)
N2—Cu—N1^i^	94.20 (14)	C8—C7—H7	119.8
N2^i^—Cu—N1^i^	85.80 (14)	C6—C7—H7	119.8
N1'^i^—Cu—N1^i^	21.80 (14)	C7—C8—C9	119.3 (4)
N1'—Cu—N1^i^	158.20 (15)	C7—C8—H8	120.3
N1—Cu—N2	85.80 (14)	C9—C8—H8	120.3
N2^i^—Cu—N1	94.20 (14)	C10—C9—C8	120.3 (3)
N1'^i^—Cu—N1	158.20 (15)	C10—C9—H9	119.9
N1'—Cu—N1	21.80 (14)	C8—C9—H9	119.9
N1^i^—Cu—N1	180	C9—C10—C5	120.1 (3)
N2—Cu—O1^i^	89.99 (9)	C9—C10—H10	119.9
N2^i^—Cu—O1^i^	90.01 (9)	C5—C10—H10	119.9
N1'^i^—Cu—O1^i^	82.27 (10)	C3—C11—C12	116.4 (3)
N1'—Cu—O1^i^	97.73 (10)	C3—C11—H11A	108.2
N1^i^—Cu—O1^i^	103.76 (15)	C12—C11—H11A	108.2
N1—Cu—O1^i^	76.24 (15)	C3—C11—H11B	108.2
N2^i^—Cu—O1	89.99 (9)	C12—C11—H11B	108.2
N1'^i^—Cu—O1	97.73 (10)	H11A—C11—H11B	107.3
N1'—Cu—O1	82.27 (10)	N1^i^—C12—C14	118.9 (3)
N1^i^—Cu—O1	76.24 (15)	N1^i^—C12—C11	98.7 (3)
O1^i^—Cu—O1	180.0	C14—C12—C11	111.8 (3)
O3—Cl1—O2	111.7 (2)	N1^i^—C12—C13	106.9 (3)
O3—Cl1—O4	109.3 (2)	C14—C12—C13	110.0 (3)
O2—Cl1—O4	108.75 (18)	C11—C12—C13	109.8 (3)
O3—Cl1—O1	109.20 (19)	C14—C12—N1'^i^	91.3 (3)
O2—Cl1—O1	109.04 (16)	C11—C12—N1'^i^	117.8 (3)
O4—Cl1—O1	108.80 (16)	C13—C12—N1'^i^	114.8 (3)
Cl1—O1—Cu	132.88 (15)	C15—C13—C12	115.0 (3)
C12^i^—N1—C1	115.5 (3)	C15—C13—H13A	108.5
C12^i^—N1—Cu	115.9 (3)	C12—C13—H13A	108.5
C1—N1—Cu	106.3 (3)	C15—C13—H13B	108.5
C12^i^—N1—H1	106.1	C12—C13—H13B	108.5
C1—N1—H1	106.1	H13A—C13—H13B	107.5
Cu—N1—H1	106.1	C12—C14—H14A	109.5
C3—N2—C2	123.2 (3)	C12—C14—H14B	109.5
C3—N2—Cu	125.7 (2)	H14A—C14—H14B	109.5
C2—N2—Cu	110.97 (19)	C12—C14—H14C	109.5
C2—C1—N1	106.7 (3)	H14A—C14—H14C	109.5
C2—C1—H1A	110.4	H14B—C14—H14C	109.5
N1—C1—H1A	110.4	C20—C15—C16	118.6 (3)
C2—C1—H1B	110.4	C20—C15—C13	120.3 (3)
N1—C1—H1B	110.4	C16—C15—C13	121.1 (3)
H1A—C1—H1B	108.6	C17—C16—C15	120.6 (3)
N2—C2—C1'	106.8 (2)	C17—C16—H16	119.7
N2—C2—C1	110.5 (3)	C15—C16—H16	119.7
N2—C2—H2A	109.5	C16—C17—C18	120.6 (3)
C1'—C2—H2A	137.7	C16—C17—H17	119.7
C1—C2—H2A	109.5	C18—C17—H17	119.7
N2—C2—H2B	109.5	C19—C18—C17	119.1 (3)
C1'—C2—H2B	78.6	C19—C18—H18	120.5
C1—C2—H2B	109.5	C17—C18—H18	120.5
H2A—C2—H2B	108.1	C18—C19—C20	120.3 (3)
N2—C2—H2C	110.4	C18—C19—H19	119.9
C1'—C2—H2C	110.4	C20—C19—H19	119.9
C1—C2—H2C	76.6	C15—C20—C19	120.7 (3)
H2B—C2—H2C	133.9	C15—C20—H20	119.6
N2—C2—H2D	110.4	C19—C20—H20	119.6
C1'—C2—H2D	110.4	C1'—N1'—C12^i^	110.16 (17)
C1—C2—H2D	133.4	C1'—N1'—Cu	95.92 (8)
H2A—C2—H2D	76.1	C12^i^—N1'—Cu	114.78 (19)
H2C—C2—H2D	108.6	C1'—N1'—H1'	111.7
N2—C3—C4	125.4 (3)	C12^i^—N1'—H1'	111.7
N2—C3—C11	119.8 (3)	Cu—N1'—H1'	111.7
C4—C3—C11	114.8 (3)	C2—C1'—N1'	104.57 (17)
C3—C4—C5	108.8 (2)	C2—C1'—H1'1	110.8
C3—C4—H4A	109.9	N1'—C1'—H1'1	110.8
C5—C4—H4A	109.9	C2—C1'—H1'2	110.8
C3—C4—H4B	109.9	N1'—C1'—H1'2	110.8
C5—C4—H4B	109.9	H1'1—C1'—H1'2	108.9
			
O3—Cl1—O1—Cu	124.9 (2)	C3—C4—C5—C6	−85.3 (4)
O2—Cl1—O1—Cu	2.5 (3)	C3—C4—C5—C10	92.6 (3)
O4—Cl1—O1—Cu	−115.9 (2)	C10—C5—C6—C7	−1.5 (5)
N2—Cu—O1—Cl1	−43.1 (2)	C4—C5—C6—C7	176.4 (3)
N2^i^—Cu—O1—Cl1	136.9 (2)	C5—C6—C7—C8	0.3 (5)
N1'^i^—Cu—O1—Cl1	−141.1 (2)	C6—C7—C8—C9	0.9 (5)
N1'—Cu—O1—Cl1	38.9 (2)	C7—C8—C9—C10	−0.9 (5)
N1^i^—Cu—O1—Cl1	−137.4 (2)	C8—C9—C10—C5	−0.3 (5)
N1—Cu—O1—Cl1	42.6 (2)	C6—C5—C10—C9	1.5 (5)
N2—Cu—N1—C12^i^	151.7 (3)	C4—C5—C10—C9	−176.4 (3)
N2^i^—Cu—N1—C12^i^	−28.3 (3)	N2—C3—C11—C12	−48.0 (4)
N1'^i^—Cu—N1—C12^i^	−107.5 (5)	C4—C3—C11—C12	130.8 (3)
N1'—Cu—N1—C12^i^	72.5 (5)	C3—C11—C12—N1^i^	80.6 (4)
O1^i^—Cu—N1—C12^i^	−117.3 (3)	C3—C11—C12—C14	−45.5 (4)
O1—Cu—N1—C12^i^	62.7 (3)	C3—C11—C12—C13	−167.9 (3)
N2—Cu—N1—C1	21.9 (3)	C3—C11—C12—N1'^i^	58.3 (4)
N2^i^—Cu—N1—C1	−158.1 (3)	N1^i^—C12—C13—C15	164.9 (3)
N1'^i^—Cu—N1—C1	122.7 (4)	C14—C12—C13—C15	−64.7 (4)
N1'—Cu—N1—C1	−57.3 (4)	C11—C12—C13—C15	58.7 (4)
O1^i^—Cu—N1—C1	112.9 (3)	N1'^i^—C12—C13—C15	−165.9 (3)
O1—Cu—N1—C1	−67.1 (3)	C12—C13—C15—C20	−96.7 (4)
N1'^i^—Cu—N2—C3	30.0 (3)	C12—C13—C15—C16	83.1 (4)
N1'—Cu—N2—C3	−150.0 (3)	C20—C15—C16—C17	0.5 (5)
N1^i^—Cu—N2—C3	8.4 (3)	C13—C15—C16—C17	−179.3 (3)
N1—Cu—N2—C3	−171.6 (3)	C15—C16—C17—C18	−0.9 (5)
O1^i^—Cu—N2—C3	112.2 (3)	C16—C17—C18—C19	0.2 (5)
O1—Cu—N2—C3	−67.8 (3)	C17—C18—C19—C20	1.0 (5)
N1'^i^—Cu—N2—C2	−154.7 (2)	C16—C15—C20—C19	0.7 (4)
N1'—Cu—N2—C2	25.3 (2)	C13—C15—C20—C19	−179.5 (3)
N1^i^—Cu—N2—C2	−176.3 (2)	C18—C19—C20—C15	−1.5 (5)
N1—Cu—N2—C2	3.7 (2)	N2—Cu—N1'—C1'	−47.47 (8)
O1^i^—Cu—N2—C2	−72.5 (2)	N2^i^—Cu—N1'—C1'	132.53 (8)
O1—Cu—N2—C2	107.5 (2)	N1^i^—Cu—N1'—C1'	−128.9 (4)
C12^i^—N1—C1—C2	−172.4 (4)	N1—Cu—N1'—C1'	51.1 (4)
Cu—N1—C1—C2	−42.3 (4)	O1^i^—Cu—N1'—C1'	41.44 (6)
C3—N2—C2—C1'	−176.9 (3)	O1—Cu—N1'—C1'	−138.56 (6)
Cu—N2—C2—C1'	7.7 (3)	N2—Cu—N1'—C12^i^	−162.9 (2)
C3—N2—C2—C1	145.9 (3)	N2^i^—Cu—N1'—C12^i^	17.1 (2)
Cu—N2—C2—C1	−29.6 (3)	N1^i^—Cu—N1'—C12^i^	115.6 (4)
N1—C1—C2—N2	47.6 (5)	N1—Cu—N1'—C12^i^	−64.4 (4)
N1—C1—C2—C1'	−42.7 (3)	O1^i^—Cu—N1'—C12^i^	−74.0 (2)
C2—N2—C3—C4	5.9 (5)	O1—Cu—N1'—C12^i^	106.0 (2)
Cu—N2—C3—C4	−179.3 (2)	N2—C2—C1'—N1'	−48.2 (2)
C2—N2—C3—C11	−175.4 (3)	C1—C2—C1'—N1'	53.7 (4)
Cu—N2—C3—C11	−0.6 (4)	C12^i^—N1'—C1'—C2	−176.1 (3)
N2—C3—C4—C5	106.0 (4)	Cu—N1'—C1'—C2	64.78 (16)
C11—C3—C4—C5	−72.8 (3)		

Symmetry codes: (i) −*x*+1, −*y*+1, −*z*+1.

### Hydrogen-bond geometry (Å, °)

**Table:**

*D*—H···*A*	*D*—H	H···*A*	*D*···*A*	*D*—H···*A*
N1—H1···O1^i^	0.88	2.39	2.940 (5)	121
N1'—H1'···O2	0.88	2.29	3.104 (4)	153

Symmetry codes: (i) −*x*+1, −*y*+1, −*z*+1.
